# Quality Improvement to Sustain Age-Friendly Practice: The MHWP Medication Workflow

**DOI:** 10.1093/geroni/igaf122.273

**Published:** 2025-12-31

**Authors:** Emily Peron, Patricia Slattum, Krista Donohe, Elvin Price, Rachel Regal

**Affiliations:** Virginia Commonwealth University School of Pharmacy, Richmond, Virginia, United States; Virginia Commonwealth University College of Health Professions, Richmond, Virginia, United States; Virginia Commonwealth University School of Pharmacy, Richmond, Virginia, United States; Virginia Commonwealth University School of Pharmacy, Richmond, Virginia, United States; Virginia Commonwealth University, Richmond, Virginia, United States

## Abstract

The Mobile Health and Wellness Program (MHWP) gained recognition through the Institute for Healthcare Improvement as an Age-Friendly Health System--Committed to Care Excellence (Level-2) in 2020 by demonstrating reliable practice assessing and acting on the 4Ms of age-friendly care in a community-based health and wellness program. In partnership with the Virginia Geriatric Education Center Geriatrics Workforce Enhancement Program (GWEP), workflows for each of the Ms undergo a quality improvement process–Plan-Do-Study-Act (PDSA)--to better support program participants, faculty and student learners. Workflows include assessment followed by offering education, resources, and referrals for care and services. The medication workflow included medication reconciliation and screening for medication nonadherence and medication-related problems, including use of high-risk medications. Using PDSA, data collected in REDCap to document participant encounters related to the medication workflow was reviewed by the MHWP faculty. Engaging pharmacy students (McFarlane Scholars) in the process, five focus groups were conducted with MHWP participants to gather their perspectives on a medication self-management skills building approach to medication review. A revised workflow replaced screening for medication-related problems with an age-friendly focused medication review and skill building for medication self-management. The revised workflow was presented to MHWP faculty for additional feedback. The revised workflow will be implemented, including training for faculty and student learners, followed by re-assessment of data collected from case notes of participant encounters. Continuous quality improvement is a key component of sustainable age-friendly practice. Engaging students creates a rich learning opportunity for future healthcare professionals.

